# Chronic Subordinate Colony Housing Paradigm: A Mouse Model to Characterize the Consequences of Insufficient Glucocorticoid Signaling

**DOI:** 10.3389/fpsyt.2015.00018

**Published:** 2015-02-23

**Authors:** Dominik Langgartner, Andrea M. Füchsl, Nicole Uschold-Schmidt, David A. Slattery, Stefan O. Reber

**Affiliations:** ^1^Laboratory for Molecular Psychosomatics, Clinic for Psychosomatic Medicine and Psychotherapy, University of Ulm, Ulm, Germany; ^2^Laboratory of Molecular and Cellular Neurobiology, Department of Behavioural and Molecular Neurobiology, University of Regensburg, Regensburg, Germany; ^3^Department of Behavioural and Molecular Neurobiology, University of Regensburg, Regensburg, Germany

**Keywords:** chronic psychosocial stress, chronic subordinate colony housing, somatic and affective disorders, decreased glucocorticoid signaling, hypocorticism

## Abstract

Chronic, in particular chronic psychosocial, stress is a burden of modern societies and known to be a risk factor for numerous somatic and affective disorders (in detail referenced below). However, based on the limited existence of appropriate, and clinically relevant, animal models for studying the effects of chronic stress, the detailed behavioral, physiological, neuronal, and immunological mechanisms linking stress and such disorders are insufficiently understood. To date, most chronic stress studies in animals employ intermittent exposure to the same (homotypic) or to different (heterotypic) stressors of varying duration and intensity. Such models are only of limited value, since they do not adequately reflect the chronic and continuous situation that humans typically experience. Furthermore, application of different physical or psychological stimuli renders comparisons to the mainly psychosocial stressors faced by humans, as well as between the different stress studies almost impossible. In contrast, rodent models of chronic psychosocial stress represent situations more akin to those faced by humans and consequently seem to hold more clinical relevance. Our laboratory has developed a model in which mice are exposed to social stress for 19 continuous days, namely the chronic subordinate colony housing (CSC) paradigm, to help bridge this gap. The main aim of the current review article is to provide a detailed summary of the behavioral, physiological, neuronal, and immunological consequences of the CSC paradigm, and wherever possible relate the findings to other stress models and to the human situation.

## Introduction

### The stress concept

In the nineteenth century, the French physiologist Claude Bernard (1813–1878) noticed that relative constancy of the internal environment is critical for the functional integrity of an organism. Later, in his “emergency concept”, Walter Cannon (1871–1945) described the disruption of this internal equilibrium, thereafter referred to as homeostasis (1), by fear- or rage-induced “fight or flight” reactions. In 1936, it was Hans Selye (1907–1982), who first defined stress, and the stress response, as “the non-specific response of the body to any physical demand” (2), and made the distinction between “stress” and the “stressor” (3). According to him, “stressors” are defined as specific challenges that cause a physiological “stress” response (3). Until now, an overwhelming number of studies have focused on the physiological, in particular neuroendocrine, and behavioral consequences of an acute stress response, which are, in general, well understood.

Thus, it is commonly accepted that the physiological and behavioral responses to acute stressors are adaptive, and important to reinstate body homeostasis [(4–6); for review see (7, 8)]. While physical stressors are, thereby, defined as external challenges to homeostasis, psychological stressors are stated as the anticipation, justified or not, of a challenge to homeostasis (9). In contrast, repeated or chronic stressor exposure over several weeks or months, and the prolonged attempt of the body to reinstate homeostasis during this time – a process referred to as allostasis [for review see (5, 10)] – is thought to result in alterations of numerous body and brain systems, finally resulting in a disease state [(11, 12); for review see (5)]. However, although chronic stress-induced alterations in neuroendocrine, emotional, and immune parameters are likely to play a major role in the etiology of numerous diseases including anxiety and depressive disorders, chronic inflammatory disorders, or cancer [(13–21); for review see (22–27)], the detailed underlying mechanisms are less well understood due, at least in part, to the shortage of appropriate animal models.

#### Physiological responses to an acute stressor

In response to any acute stressor, two major stress systems become activated, namely the autonomic nervous system, especially its sympathetic (SNS) branch, and the hypothalamo–pituitary–adrenocortical (HPA) axis. Stimulation of these emergency systems, which differ in both their time course and processing, reflects the body’s attempt to deal with the immediate situation and to restore homeostasis [for review see (5, 10)]. Activation of the SNS occurs rapidly, within seconds, via exclusively neuronal pathways originating in the thoracolumbal regions of the spinal cord (splanchnic nerve), and results in the release of adrenaline from chromaffin cells of the adrenal medulla into the blood. Elevated adrenaline levels in the circulation act in synergy with an increased sympathetic noradrenergic innervation of essentially all organs in the body [referenced in (28, 29)]. As a result, cardiovascular and catabolic functions are promoted, and processes not vital in the immediate situation, such as anabolic processes and digestion, are inhibited.

In addition to the SNS, there is a slightly delayed activation of the HPA axis in response to acute stressors. The stimulation of the HPA axis is triggered by the secretion of corticotropin-releasing hormone (CRH) and arginine vasopressin (AVP) from parvocellular neurons of the paraventricular nucleus (PVN) of the hypothalamus into the portal blood stream of the pituitary stalk. CRH and AVP promote the synthesis and the secretion of adrenocorticotropic hormone (ACTH) from anterior pituitary corticotroph cells into the peripheral blood, which, in turn, stimulates cortical cells of the adrenal gland to produce and secrete glucocorticoids [GCs; cortisol in humans, corticosterone (CORT) in rats and mice] into the circulation. Within minutes of activation, termination of an acute HPA axis response is achieved by efficient negative feedback inhibition via GC acting at GC receptors and mineralocorticoid receptors at several brain levels (30). The degree and temporal dynamics of HPA axis activation are strongly dependent on the quality, intensity, and duration of the acute stressor (11, 31, 32). In addition, the acute neuroendocrine stress response was shown to be dependent on the time of day (33–35), age of an individual [(36); for review see (37)], reproductive status of an individual [e.g., in the peripartum period (38–40)], genetic background (41–45), and stressor exposure during life history (46, 47).

Taken together, stressor-induced activation of the SNS and HPA axis contribute to the restoration of the “internal equilibrium” by rapid mobilization of metabolic resources (glucose, oxygen); processes that are adaptive and essential for survival.

#### Behavioral responses to an acute stressor

In addition to, and facilitated by, the rapid activation of physiological systems (SNS, HPA axis) in response to an acute stressor, there is an instant behavioral response, such as arousal, anxiety/fear, or aggression. This behavioral flexibility is regulated via activation of a number of brain regions, including cortical areas, limbic regions, and the brainstem (48–50). A region of particular importance is the lateral septal area, which is thought to segregate the autonomic, neuroendocrine, and behavioral responses (51). In humans, the behavioral (emotional) response to acute stressor exposure is an important measure of mental health. It is mainly quantified retrospectively via questionnaires (52–54) or by analyzing behavioral patterns known to be linked with distress during, for instance, public speaking (55–57). In laboratory animal models, a variety of behavioral tests have been established in order to quantify signs of arousal (e.g., measurement of homecage activity/locomotion), fear and anxiety-/social anxiety-related behavior [e.g., novelty-supressed feeding, shock pole burying, elevated plus-maze (EPM) test, light–dark box (LDB) test, open arm exposure test, open field test, social preference/avoidance test (SPAT), elevated platform (EPF) exposure, resident-intruder test, Vogel test, 4-plate test, marble burying, stress-induced hyperthermia, contextual/cued fear conditioning, acoustic startle], learning deficits (e.g., Morris water maze, Y/T-maze, holeboard, Barnes maze), anhedonia (e.g., sucrose preference test, progressive ratio responding, psychostimulant-induced hyperactivity, female urine sniffing test), memory skills (contextual-/cued fear conditioning), aggression (resident-intruder test), and active versus passive stress coping strategies [e.g., forced swim test (FST), tail suspension test (TST), learned helplessness] [for reviews dealing with these, and additional tests to assess such behaviors see (58–65)]. Similar to the physiological stress response, the behavioral stress response is strongly dependent on the time of day of stressor exposure (33), quality, intensity, and duration of the stressor (66–70), as well as the genetic and environmental background of the organism (41, 46, 47, 71–74).

### Chronic stress in humans

#### Mal-adaptive consequences of chronic stressor exposure

While the acute stressor-induced changes described in the sections above are adaptive, chronic activation of the two stress systems poses an acknowledged risk factor for numerous disorders, including somatic disorders, like cardiovascular diseases [(75–79); for review see (80)], chronic fatigue syndrome (81), fibromyalgia (82), bronchial asthma (83, 84), atopic dermatitis [for review see (85)], arthritis [(86); for review see (87)], inflammatory bowel disease (IBD) [(13, 14, 16, 18, 20, 21); for review see (22, 25, 26)], stomach ulcers (86), diarrhea and digestive problems (86, 88), chronic pelvic and abdominal pain (86, 88), infections (86, 88–90), headaches (86, 88), impaired wound healing (91–93), cancerogenesis [(17); for review see (27, 94)], as well as affective disorders, like anxiety disorders and depression [(95–98); for review see (24, 58, 60, 99, 100)]. While the underlying etiology of these diseases are not fully understood, due at least in part to a lack of animal models, chronic stress-induced dysregulation of almost all psycho-neuro-immunological systems including the HPA axis, the autonomic nervous system, the immune and cardiovascular systems, and emotional and cognitive brain circuits is highly likely to contribute to the complex, and multifactorial, etiology of such disorders. On closer inspection, one mechanism that appears to be common throughout all of these diseases and chronic stress models is altered GC signaling.

#### The link between chronic stress and impaired GC signaling

Raison and Miller defined decreased GC signaling as “any state in which the potential of GC is inadequate to restrain relevant stress-responsive systems”. Such inappropriate GC signaling can be the result of decreased hormone bioavailability (hypocorticism), attenuated GC sensitivity/enhanced GC resistance of target cells, or the combination of both [for review see (101)]. Although HPA axis hyperactivity (hypercorticism) has been generally linked to prolonged, or chronic, stressor exposure, there is accumulating evidence for additional, even opposite alterations [for review see (23)]. In this respect, chronic stress-induced hypocorticism regained consideration after being more or less ignored up until the beginning of the 2000s. For example, Friedman and colleagues in the early 1970s described decreased plasma and urinary cortisol levels in parents of children suffering from neoplastic disease, with a paradoxical decrease during periods of heightened stress (102). Lower basal GC levels were further reported in high work load employees (103) and patients suffering from post-traumatic stress disorder [for review see (104)]. While elevated basal GC levels have been repeatedly linked to stress-related disorders like major depression [(105); for review see (100, 106)], the overall GC signaling in these patients has been shown to be decreased both *in vivo* and *in vitro* as a consequence of GC insensitivity [for review see (106, 107)]. Taken together, this growing body of evidence has led to greater acceptance of the idea that chronic stress experiences in adulthood result in an insufficient GC signaling.

In addition, chronic stress experienced early in life, like loss of parents, emotional neglect, maltreatment, or abuse have also been linked to a reduced GC signaling capacity in humans. In this context, it has been shown that women maltreated during early life exhibited lower basal and ACTH-induced plasma cortisol levels, an effect that was probably mediated by adrenal dysregulation (108, 109). However, whether the reduction in the overall GC signaling poses a central and causal mechanism by which chronic stress causes the variety of somatic and affective disorders described above is still unknown, but likely.

#### Many stress-related disorders are linked to a decrease in GC signaling

Although a causal involvement still has to be proven, as stated above, several chronic stress-related pathologies have been shown to be concurrent with reduced GC signaling in a growing number of studies. For example, hypocorticism has been described in patients suffering from burnout and chronic fatigue syndrome, fibromyalgia, chronic pelvic pain, and geriatric depression [(105, 110–112; for review see (23)]. Low levels of plasma GC have been further reported when suffering from inflammatory disorders, including rheumatoid arthritis [for review see (113)] or asthma (114). In line with this, elevated levels of pro-inflammatory cytokines have been reported in patients suffering from acute GC deficiency after surgical removal of adrenal cortical tissue (115). Moreover, it has recently been shown that obese women have lower cortisol levels during pregnancy (116). Interestingly, based on human and animal studies, it has been hypothesized that the onset of IBD might be associated with hypo- rather than hypercorticism [for review see (117, 118)]. This is further supported by a recent finding showing an impaired HPA axis reactivity in 25% of Crohn’s patients during exposure to the ultra-low dose ACTH test (119). In addition, a positive correlation between plasma cortisol levels and the time patients were off steroid treatment has recently been described (120). Finally, Rodriguez and coworkers speculated that a down-regulated cortisol response to intero- and exteroceptive stressors might predispose patients suffering from irritable bowel syndrome to chronic inflammatory conditions, such as asthma, rheumatoid arthritis, or IBD (121).

Besides hypocorticism, GC resistance has been speculated to contribute to the reduced GC signaling and the pro-inflammatory immune shift in patients suffering from chronic stress-related pathologies (122). As mentioned above, the disorder that best fits this context is major depression [for review see (101)], as patients show a reduced response to GC both *in vivo* and *in vitro* [for review see (106, 107)], which is believed to be mediated, at least in part, by decreased GC receptor expression and/or functionality [(123–125); for review see (107)]. GC resistance has further been diagnosed in a subset of patients suffering from typically chronic inflammatory disorders like ulcerative colitis and Morbus Crohn [(126); for review see (127)], as well as rheumatoid arthritis (128).

To causally demonstrate that chronic psychosocial stress promotes the development of, at least some, somatic and affective disorders via a reduction in overall GC signaling, it is necessary to have appropriate animal stress models, which mimic the human situation in an adequate way. Thus, animal models are warranted that are of chronic psychosocial nature, to show face validity, and cause both somatic and affective disorders, as well as result in a reduced GC signaling (ideally both hypocorticism and decreased GC sensitivity), to provide predictive validity. Given that the vast majority of somatic and affective disorders are multifactorial diseases, for which the underlying etiological factors are only poorly understood, most of the animal models fail to satisfy construct validity. However, if insufficient GC signaling is indeed causally involved in the development of many such diseases, animal models resulting in either hypocorticism or a decreased GC sensitivity, or both, could be considered primarily as models displaying construct validity.

In the following paragraphs, we will in detail describe the chronic subordinate colony housing (CSC) paradigm, which fulfills all the criteria outlined above and, thus, represents an adequate and preclinically validated (face and predictive validity) animal model to investigate the underlying mechanisms related to chronic psychosocial stress-induced impaired GC signaling and its involvement in somatic and affective disorders.

## Chronic Subordinate Colony Housing

### General description and experimental details

The CSC paradigm combines chronic, psychological, and social aspects of stress and, thus, represents a highly potent animal model to mimic the type of health compromising stressors faced by humans (high face validity). CSC further promotes development of both somatic and affective disorders, results in a reduced GC signaling (high predictive/construct validity) and, thus, provides a powerful experimental tool to study the mechanisms underlying several relevant stress-induced pathologies. Notably, given that the CSC paradigm is simply based on the fact that male mice instinctively establish a certain hierarchical structure within their colony, it additionally resembles the natural way of life of a male mouse in the wild [(129); for review see (9)]. However, before we detail the physiological, immunological, and behavioral consequences of the CSC paradigm unraveled to date, we will briefly introduce the experimental details of this chronic psychosocial stress model.

**Figure 1:**
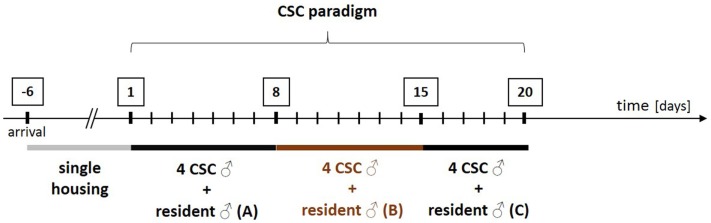
**Schematic illustration of the experimental design of the chronic subordinate colony housing (CSC) paradigm**. After arrival, all experimental male mice are housed singly for 1 week before they are assigned to the single-housed control (SHC) or the CSC group in a weight matched manner. In order to induce chronic psychosocial stress, CSC mice are housed together with a larger dominant male for 19 consecutive days. In detail, four experimental CSC mice are introduced into the homecage (polycarbonate observation cage; 38 cm × 22 cm × 35 cm) of resident A on day 1 of CSC, resulting in immediate subordination of the four intruder CSC mice. The latter are then housed together with this dominant resident (A) for eight consecutive days. On day 8, and again on day 15, of CSC, the four experimental CSC mice are transferred into the homecages of resident B (day 8) and resident C (day 15) to avoid habituation. Before the CSC procedure, the future dominant males are tested for their aggressive behavior. Males that start to injure their opponents by excessive and harmful bites during testing are not used.

During CSC (for details see Figure 1), experimental mice (CSC mice) live in chronic subordination to a dominant resident mouse for 19 consecutive days (130). In detail, four CSC mice are put into the homecage of a larger male resident mouse on day 1 of the CSC paradigm, resulting in immediate subordination of the four intruder CSC mice. To avoid habituation, all four CSC mice are transferred into the homecage of a novel larger male resident mouse on days 8 and 15.

**Figure 2:**
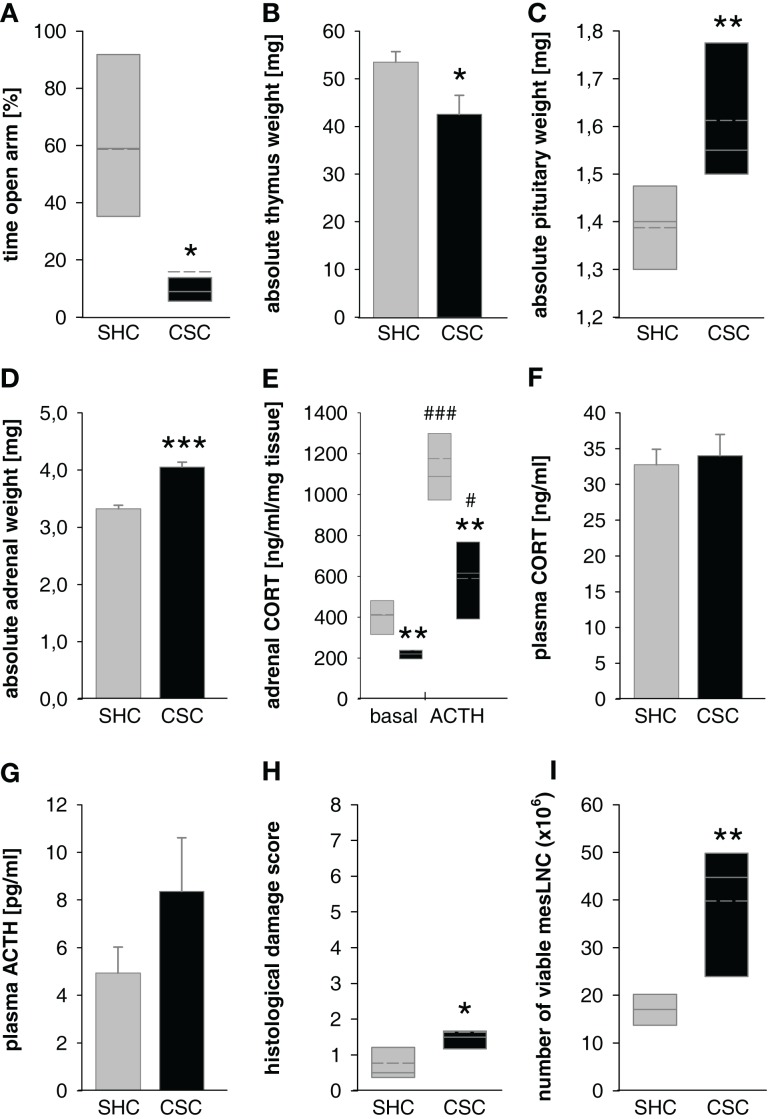
**Effects of 19 days of CSC exposure on behavioral, physiological, and immunological parameters**. Dominant resident male mice originated from the CD1 strain, CSC mice from the C57BL/6 strain. CSC exposure resulted in increased anxiety-related behavior measured on the elevated plus-maze [EPM; **(A)**] without affecting locomotor activity (data not shown), decreased thymus weight **(B)**, increased pituitary weight **(C)**, increased adrenal weight **(D)**, reduced adrenal *in vitro* ACTH sensitivity **(E)**, unaffected basal morning plasma corticosterone [CORT; **(F)**], a tendency toward increased basal morning plasma adrenocorticotropic hormone [ACTH; **(G)**], increased histological damage in colonic tissue **(H)**, and increased number of viable mesenteric lymph node cells [mesLNC; **(I)**]. 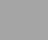 SHC (*n* = 7–8); ■ CSC (*n* = 7–8). Parametric data are represented as mean + SEM. Non-parametric data are represented in box-plot diagrams. Boxes signify the upper and lower quartiles, the median is represented as solid line, and the mean as dashed line within each box. *P < 0.05, **P < 0.01, ***P < 0.001 versus respective SHC; ^#^*P* < 0.05, ^###^*P* < 0.001 versus respective basal values.

All resident males are tested before CSC housing for their aggressive behavior and males that injure their opponents by excessive aggression (e.g., harmful bites) are not used. Notably, although this procedure strongly reduces the number of bite wounds delivered by the residents during CSC exposure, it does not 100% prevent them. As a matter of routine, the subordinate position of each CSC mouse is confirmed by behavioral analysis of the first 30 min after setting up the CSC colonies on days 1, 8, and 15 (131). Resident males reliably (>99% of CSC colonies) obtain the dominant position by displaying offensive behaviors toward the CSC mice, such as chasing, mounting, or attacking their four cage mates (131). In contrast, CSC mice can be considered as “subordinates” based on their defensive behaviors, including flight and submissive upright (131). So far, the CSC model has reproducibly been shown to work in different mouse strains, namely, C57BL/6 mice (130), BALB/c mice (132), and CD1 mice (41). Moreover, CSC effects are independent from the background of the residents, as the physiological, immunological, and behavioral consequences of the CSC paradigm are comparable using either C57BL/6 (130) or the male offspring of CD1 female mice [bred at the Max Planck Institute of Psychiatry in Munich (Germany) for high anxiety-related behavior (HAB mice)] and male C57BL/6 mice as dominant animals (133, 134). Recent own unpublished data reveal that using male CD1 mice as residents allows reliable reproduction of known CSC effects (see Figure 2).

An important issue for the design of chronic psychosocial stress paradigms is the choice of adequate same-aged controls, with single housing [single-housed control (SHC) mice] or group housing [group-housed control (GHC) mice] being widely used options. For CSC experiments, we employ SHC mice based on own data indicating that group housing *per se* poses a stressful condition for male mice. Surprisingly, similar physiological and behavioral alterations after 3 weeks of GHC or CSC were observed, leading us to believe that the novel hierarchy formed by GHC mice is as almost as stressful as being subordinated by a dominant resident. For example, lower body weight gain and increased state anxiety were found in both CSC and GHC compared with SHC mice (135). In detail, the number and time of head dips and distance traveled on the open arm of the EPM were reduced in both CSC and GHC compared with SHC (135) mice;  parameters related to risk assessment, anxiety, exploratory (136), and locomotor behavior (137), respectively. Given that isolation has been shown to lack effects on stress-related immune and/or endocrine functions in male mice by other stress laboratories (138, 139), single housing seems to be the most appropriate non-stressful control condition in non-sibling male mice. In line with this, Blanchard et al. [for review see (140)] and Palanza [for review see (141)] proposed that isolation is more stressful for female mice, while social grouping is more stressful for male mice. In males, any kind of group housing is likely to be accompanied by the establishment of subtle hierarchies with the result that in each cage dominant and more or less subordinate cage mates can be found (135).

### CSC-induced consequences

#### Endocrine changes

##### Adrenal gland, pituitary, and acute stress reactivity

The CSC paradigm has been shown to result in profound and reproducible physiological changes, including a significant (41, 46, 130, 133, 142) and long-lasting (at least until day 8 after termination of CSC) (70) enlargement of the adrenal glands. This increase in absolute adrenal mass is mediated by cell hyperplasia (133), without alterations in adrenal cholesterol delivery pathways [cortical lipid droplets; protein expression of hormone-sensitive lipase, 3-hydroxy-3-methylglutaryl coenzyme A reductase, and low-density lipoprotein receptor, with the exception of the scavenger receptor class B type 1 protein, which was increased following CSC exposure (133)]. Notably, in an adrenocortical cell the CORT precursor molecule cholesterol can among others be derived from (i) hormone-sensitive lipase-mediated hydrolyzation of cholesteryl esters, stored in lipid droplets within the cytoplasm of mostly zona fasciculata cells [for review see (143)], (ii) hormone-sensitive lipase-mediated hydrolyzation of cholesteryl esters “selectively” taken up from high-density lipoproteins and low-density lipoprotein via the scavenger receptor class B type 1 [(144, 145); for review see (146–148)], (iii) lysosomal acid lipase-mediated hydrolyzation of cholesteryl esters from low-density lipoprotein taken up endocytotically via the low-density lipoprotein receptor (149), and (iv) endogenous *de novo* synthesis from acetyl coenzyme A via the 3-hydroxy-3-methylglutaryl coenzyme A reductase [for review see (150)]. Thus, given the elevated adrenal weight following 19 days of CSC, the molecular and cellular changes reported above in CSC versus SHC mice at the level of the adrenal glands strongly suggest an enhanced adrenal availability and/or mobilization capacity of the CORT precursor molecule cholesterol and, consequently, an increased adrenal functionality in CSC versus SHC mice. In line, analysis of plasma high-density lipoprotein cholesterol and low-density lipoprotein cholesterol revealed increased levels of the latter in CSC versus SHC mice. Similarly, a comparable or even increased relative expression of melanocortin 2 receptor protein and melanocortin 2 receptor accessory protein mRNA, as well as of steroidogenic acute regulatory protein mRNA, side-chain cleavage enzyme mRNA, 11β-hydroxylase and aldosterone-synthase mRNA – the latter enzymes are known to be essential in the progress of CORT synthesis from its precursor cholesterol [for review see (151, 152)] and to be controlled by ACTH signaling [for review see (153, 154)] – support the idea of an overall increased adrenal functionality following CSC exposure.

In confirmation of this hypothesis, mice exposed to 19 days of CSC indeed show exaggerated plasma CORT concentrations, as well as an increased adrenal CORT content, when killed 5 min following termination of a mild acute heterotypic stressor (EPF) at the beginning of the light phase, despite the increase in plasma ACTH concentrations not differing from EPF-exposed SHC mice (142). Notably, when exposed to a more severe acute heterotypic stressor, i.e., 6-min of forced swimming, CSC mice even show an exaggerated ACTH response compared with SHC mice (155), likely to further enhance HPA axis reactivity toward a novel and severe enough heterotypic challenge in CSC mice. A facilitated ACTH response to a novel heterotypic challenge is thereby in line with other chronic stress studies [(156); for review see (157)].

In line with what we found at the adrenal level, the increased capability of the CSC pituitary gland to produce and secret ACTH is mediated at least partly by corticotroph cell hyperplasia (155). Non-compromised functionality of those newly formed cells was suggested by comparable relative pituitary pro-opiomelanocortin protein expression between CSC and SHC mice (155). Interestingly, the idea that during conditions of prolonged/chronic stress AVP becomes the main pituitary ACTH secretagogue [for review see (157)] is supported by unaltered relative pituitary AVP1b receptor and decreased CRH receptor 1 protein expression in CSC versus SHC mice (155). Taken together, these data suggest that these newly formed corticotrophs shift their sensitivity from CRH to AVP. Increased AVP output at the level of the PVN – as suggested by other studies dealing with repeated/chronic stressor exposure [for review see (157)] – does not appear to enhance pituitary AVP stimulation and, thus, to contribute to the increased ACTH drive in CSC mice, as the number of AVP positive parvocellular PVN neurons is comparable between CSC and SHC mice (155). In line, mRNA expression of AVP is even lower in the PVN of CSC versus SHC mice (131), while CRH mRNA is not affected (130). Furthermore, neuronal activation in the parvocellular PVN (predominantly AVP and CRH neurons) is lower in CSC versus SHC mice following acute heterotypic stressor exposure (open arm; 5 min) (135). Similarly, the contributing role of changes in pituitary negative feedback inhibition to the increased ACTH secretion in CSC mice seems to be negligible, as the dexamethasone suppression test indicated a fully functional feedback system (155). Notably, the latter finding clearly indicates that a decrease in pituitary cytoplasmic GC receptor protein expression, as seen in CSC versus SHC mice (155), cannot generally be interpreted as an impairment of negative feedback function. This is of considerable importance for the stress field in general, as in many published studies this was common practice.

In addition to the above described data assessed on day 20 of CSC (after 19 days of CSC), a time course analysis revealed that relative adrenal mass was significantly increased in stressed mice at all time-points assessed (24, 48 h, days 7, 14, 20) during the 19 days of CSC exposure (130). A more recent study, confirming the increase in relative adrenal weight following 48 h of CSC, extended these findings to demonstrate that even 10 h of CSC are sufficient to cause such changes (134). However, in contrast to relative weight, absolute adrenal weight during this initial phase of CSC was increased after 10 h, but not 48 h, of CSC exposure. Considering the reduction in body weight at both these time-points (70, 132, 134), this clearly indicates that the increase in relative adrenal weight observed following 48 h of CSC is exclusively due to changes in body weight and not to changes at the adrenal level *per se*. Given the reliable increase of absolute adrenal mass following 19 days of CSC described earlier (41, 46, 130, 133, 142), these data for the first time indicate that the adrenal glands of an organism exposed to chronic psychosocial stress enlarge during the very initial phase of stressor exposure, normalize after about 48 h of continuous challenge and, given the stressor still persists, start to enlarge again.

##### Basal plasma CORT, ACTH and noradrenaline, and GC signaling

Interestingly, these changes in absolute adrenal weight during the initial phase of CSC seem to run in parallel with the fluctuations of basal morning plasma CORT levels. Following 10 h of CSC exposure, plasma morning CORT (132, 134, 158), as well as absolute adrenal mass (134), are significantly increased, whereas following 48 h both parameters return to baseline values. Thus, it seems that reversing the early increase in adrenal mass in CSC mice (10 h) poses some kind of adaptive mechanism, contributing, together with a reduction of stimulatory adrenal input (ACTH) from the pituitary and a possibly increased CORT metabolism (134), to prevent the organism from prolonged exposure to elevated plasma CORT concentrations. This is supported by studies showing a positive correlation between plasma CORT and adrenal weight under stress conditions in rats (159, 160). Chronically elevated CORT concentrations are known to have deleterious health consequences [(161); for review see (162–164)] and to cause increased anxiety- and depressive-like behavior in rats. Moreover, as greater insights can be obtained from rodent studies, chronically high CORT levels have also been shown to affect the brain serotonergic system (165), as well as to rapidly and dramatically increase body weight gain, adiposity, plasma leptin, insulin, and triglyceride levels, and also to decrease homecage locomotion (166) when delivered via the drinking water. However, future studies are needed to clarify whether these early adaptive changes in absolute adrenal weight during CSC exposure are mediated by hyper-/hypotrophy or by hyperplasia/apoptosis of adrenal cells.

Interestingly, although absolute adrenal mass increases again subsequent to the 48 h time point, plasma morning CORT concentrations on days 7, 14, 20 stay still comparable to those of SHC mice (41, 46, 130, 142). This suggests a mechanism different from the one involved during the initial phase of chronic stressor exposure to prevent the deleterious consequences of hypercorticism in the later phases of chronic stressor exposure. Given that isolated adrenal cells (130), as well as adrenal explants (142), from mice exposed to 19 days of CSC show a reduced *in vitro* CORT release when treated with different ACTH doses, it is likely that this is implemented via a reduced ACTH sensitivity of cortical adrenal cells. Notably, adrenal ACTH sensitivity seems to be not only diminished under *in vitro* conditions, as unaffected basal morning plasma CORT in 19-day CSC mice is paralleled by elevated plasma ACTH in comparison with SHC mice (41, 155). Support for this second mechanism to play a role only after prolonged stressor exposure and not to contribute to the initial normalization of plasma CORT is provided by our finding that adrenal *in vitro* ACTH sensitivity of CSC mice was not different from SHC at the 10 and 48 h time point (134).

While the reduction in adrenal ACTH sensitivity in the presence of increased absolute adrenal mass and plasma ACTH seems to ensure normal basal morning CORT concentrations, it is likely to promote the basal evening hypocorticism detected in 19-day CSC mice (130). SHC mice were able to show the expected (167, 168) diurnal rise in plasma GC concentrations at the beginning of their active period, whereas CSC mice were not and, thus, had lower plasma CORT concentrations than respective SHC mice in the evening of day 20 of CSC (130). The resulting decline in GC signaling was further amplified by a CSC-induced reduction in GC sensitivity. The latter was described in both lipopolysaccharide-stimulated splenocytes (130) and plate-bound anti-CD3-stimulated T helper (Th) 2 cells from peripheral lymph nodes (169) of 19-day CSC compared with SHC mice. Thus, given the accumulating evidence that a reduction in GC signaling might be involved in the development of somatic and affective disorders linked with an inflammatory component [for review see (101, 170–172)], it is likely that adrenal changes seen during CSC exposure, although preventing the negative consequences of prolonged hypercorticism, contribute to the development of spontaneous colitis (130, 158, 173), hepatic inflammation (174), increased anxiety-related behavior (41, 46, 70, 130, 131, 135, 142, 173, 175), hyperactivity (70), and the increased risk of inflammation-related colorectal cancer (CRC) (176). In support, we previously showed additive effects of early life stress (repeated maternal separation) and CSC exposure on both the development of hypocorticism and on the severity of a chemically induced colitis (46). To assess, adrenalectomy with CORT replacement needs to be performed to see what CSC-induced behavioral and physiological effects remain.

Notably, as already discussed above in detail, this decreased adrenal ACTH sensitivity is not preventing CSC mice from showing an exaggerated CORT response to subsequent EPF exposure, suggesting an additional, yet unknown, factor that is enhanced in CSC mice during acute heterotypic stressor exposure, unaffected by the diurnal rhythm, which rescues the attenuated adrenal ACTH responsiveness. For example, sympathetic innervation of the adrenal medulla via the splanchnic nerve is known to play a critical role in modulating adrenocortical sensitivity to ACTH (177–179). Following activation, adrenal medullary cells secrete neurotransmitters and neuropeptides such as adrenaline/noradrenaline, neuropeptide Y, vasoactive intestinal peptide, or substance P, which may, in a paracrine manner [for review see (180, 181)], influence adrenocortical CORT secretion. Moreover, neuropeptides such as prolactin and oxytocin (OXT), which are released during various types of acute stressor exposures [(39,182); for review see (183)], act as direct CORT secretagogues (184–186). Therefore, instead of rescuing ACTH signaling, it is also possible that this unknown factor is a CORT secretagogue itself, thereby simply replacing ACTH in the process of adrenal activation during heterotypic stressor exposure. However, future studies are required to elucidate the identity of this currently unknown determinant.

In contrast to the reduction of basal adrenal cortex function, increased basal plasma noradrenaline concentrations following CSC (130) indicate an over-activated adrenal medulla and, thus, uncoupling the activity of the HPA axis and the SNS during chronic psychosocial stressor exposure. As the concerted action of steroid hormones and neurotransmitters of the SNS is crucial for optimal immunosuppression, uncoupling of the HPA axis and the SNS is likely to further promote pro-inflammatory processes (187). Thus, future studies focusing on the role of the SNS in CSC-induced pathology are warranted.

##### Summary

In summary, exposure to CSC initially (10–24 h) triggers a pronounced HPA axis response, resulting in increased absolute adrenal mass and elevated basal morning plasma GC concentrations. Following 48 h of continuous CSC exposure, basal morning GC concentrations return to basal levels again, mediated most likely by a combination of decreased stimulatory input from the pituitary, enhanced CORT metabolism, and restoration of normal adrenal mass. Interestingly, during further stressor continuation, the recurrence of rising absolute adrenal mass is not paralleled by increased (morning), but rather decreased (evening) basal plasma GC concentrations, mediated at least partly via a pronounced reduction in adrenal ACTH responsiveness.

#### Body weight changes

While decreased body weight gain has been reported in many studies investigating the effects of repeated/chronic stressor exposure (67, 188–191), other studies have reported no alteration in body weight development (192–195). In line, the effects of CSC exposure on body weight development are not fully consistent, resulting in either decreased (130, 131, 135, 169, 173, 176) or unaffected body weight gain (41, 46, 70). Therefore, while CSC seems to reliably diminish body weight gain during the initial phase of CSC exposure (46, 70, 131, 132, 134), this in some sets of mice normalizes over the final days of stressor exposure. Notably, CSC mice further gained significantly more weight in the week after stressor termination than unstressed SHC controls leading to a normalization or even increased bodyweight of CSC versus SHC mice (70). Similar findings have previously been reported following subjection to the visible burrow system (196) and repeated social defeat (194) and may represent a general phenomenon following prolonged stressor exposure. This increase in body weight after termination of chronic stress may be an adaptive mechanism for ensuring sufficient resources in preparation for subsequent stressful events. Together, these data emphasize the necessity to further investigate the link between repeated/chronic stressor exposure and changes in body weight and, suggest that caution should be exhibited when interpreting a lack of reduced body weight gain as a sign of a non-effective chronic stress paradigm.

#### Somatic disorders

In addition to the consequences on endocrine parameters and body weight development, CSC represents an established model to study the immunological consequences of chronic psychosocial stress exposure. In agreement with other chronic social stress paradigms (188, 189, 197–201), CSC causes thymic involution, first detected after 24 h (130), which is in line with the thymus atrophy reported in rats following 24 h of resident-intruder confrontations (189, 201).

##### Inflammation

Interestingly, and again in line with others (198, 202, 203), CSC causes splenomegaly (41) and reduced *in vitro* GC sensitivity in isolated and lipopolysaccharide-stimulated splenocytes (130). Given that this is paralleled by pronounced immune activation in the social disruption (SDR) paradigm (203–205), it is very likely that the systemic immune status of CSC mice is enhanced as well. GC resistance of IL-4 producing Th2 cells, a reduced number of regulatory T cells, and an increased T cell effector function, all detected in peripheral lymph nodes following 19 days of CSC exposure (169) support this idea. Moreover, CSC mice show higher hepatic tumor necrosis factor alpha, monocyte chemotactic protein 1, and heme oxygenase mRNA expression, indicating noticeable oxidative stress and hepatic inflammation (174), and develop a more severe colitis when subsequently treated with dextran-sulfate sodium (DSS, 1%, 7 days) (46, 173). The latter was indicated by increased body weight loss, inflammatory reduction of colon length, and histological damage score in CSC versus SHC mice after 8 days of DSS treatment.

Interestingly, unlike SHC, CSC mice already on the second day of DSS treatment demonstrate an increased cytokine secretion from isolated and plate-bound anti-CD3-stimulated mesenteric lymph node cells (173), suggesting chronic subordination itself to trigger the development of a colonic inflammation. In support, stimulated cytokine secretion from isolated mesenteric lymph node cells is increased in non-DSS treated CSC mice 8 days following stressor termination (173). Finally, confirming chronic subordination-induced spontaneous colitis, CSC mice display an increased histological damage score in the colon – first detectable after 14 days of CSC (130), number of colonic macrophages, dendritic, and Th cells (158), and cytokine secretion from *in vitro* stimulated mesenteric lymph node (130) and lamina propria mononuclear cells (158). Notably, comparable to the CSC paradigm, a modified version of the SDR paradigm (202–204, 206) has recently been shown to also cause mild histological colonic damage in male mice (195).

Based on the absent CORT increase in the plasma of CSC mice on the second day of DSS treatment – despite increased *in vitro* stimulated cytokine secretion from mesenteric lymph node cells at this time – we recently hypothesized that CSC-induced adrenal insufficiency contributes to the increased severity of DSS-induced colitis [for review see (118)]. In contrast, increased cytokine secretion from *in vitro* stimulated mesenteric lymph node cells of SHC mice was first detected on the eighth day of DSS treatment, and immediately paralleled by high plasma CORT concentrations (173). However, the adrenal hyper-reactivity toward heterotypic stressors in CSC versus SHC mice clearly argues against a general break down of adrenal functioning in CSC mice. Thus, it is rather likely that cytokine levels secreted from mesenteric lymph node cells *in vivo* during DSS treatment were not high enough to spill over into the systemic circulation and, thereby, activate the HPA axis until day 4 of DSS treatment in both CSC and SHC mice. In turn, a more pronounced plasma CORT increase in CSC versus SHC mice on day 8 of DSS treatment suggests elevated systemic cytokine levels in both groups, and indicates HPA axis hyper-reactivity in CSC mice also to occur in response to heterotypic stressors of an inflammatory nature, given the assumption that inflammation is severe enough to activate the HPA axis.

Support for decreased basal GC signaling – caused by basal hypocorticism and/or GC resistance (130, 169) – to promote CSC-induced aggravation of DSS-induced colitis is provided by the finding that the combination of early-life stress (maternal separation, MS; 3 h/day, from postnatal day 1–14) and 19 days of CSC during adulthood has additive effects on DSS-induced colitis. In contrast to CSC mice, which are only unable to adequately increase plasma CORT at the beginning of the dark/active phase, mice exposed to both MS and CSC suffer from hypocorticism even during the morning hours (46).

With respect to the mechanisms underlying the development of CSC-induced spontaneous colitis, assessment of several functional levels of the colon following the initial stress phase (10 h of CSC) revealed a pronounced, adrenal hormone-mediated, local immune suppression in colonic tissue; probably allowing luminal- and translocated-bacteria to proliferate without constraint (158). Immune suppression was indicated by a reduced cytokine and immunoglobulin A secretion from isolated and anti-CD3/IL-2-stimulated lamina propria mononuclear cells, a decreased percentage of CD3^+^ cells within all isolated lamina propria mononuclear cells, a decreased pro-inflammatory colonic cytokine mRNA expression, and a lower number of F4/80^+^ macrophages, CD11c^+^ dendritic cells, CD3^+^ T cells, and CD4^+^ Th cells in colonic tissue of CSC compared with SHC mice. Whether or not this effect is mediated by cortical GC or medullary catecholamines still needs further investigation. The early decrease in colonic IgA secretion in combination with an obsolescent mucosa, indicated by reduced epithelial cell proliferation and apoptosis, additionally suggested the initiation of impaired epithelial barrier functions (158). In line, 10 h of CSC resulted in a reduced/deficient mucus production of colonic epithelial cells. Surprisingly, and in contrast to early CSC-induced immune suppression, our data clearly indicated that the reduction in epithelial barrier functions was not mediated by adrenal hormones. Given that intact local immune and epithelial barrier functions are essential for the control of commensal flora, it was not surprising to detect an increased bacterial load in colonic tissue and in stool samples from CSC mice following 10 h of stressor exposure. Furthermore, experiments employing prolonged antibiotic treatment have revealed a causal role of such bacterial translocation/proliferation during the initial phase of CSC in the initiation/induction of colonic inflammation (158). However, using adrenalectomized mice, we showed that the immunosuppressive effects of high levels of adrenal hormones during the initial CSC phase were required to develop a moderate colitis into a full-blown form (158). Direct evidence showing that the over-active immune system in the later stages of CSC, i.e., when hypocorticism (46, 130) and GC resistance (130, 169) have developed, targets this elevated presence of bacterial antigens in the colonic tissue of CSC mice, leading to the observed colitis (130, 158), still needs to be provided.

##### Inflammation-related colon carcinogenesis

Given that chronic stress is an acknowledged risk factor for numerous disorders, including IBD [(13, 14, 16, 18, 20, 21); for review see (22, 25, 26)] and cancer [(17); for review see (27)], and that CRC poses one of the most serious complications in IBD patients [(207); for review see (208, 209)], it is not surprising that CSC, besides causing spontaneous colitis (130, 158), also increases the risk for inflammation-related CRC. Combining a novel colitis-related CRC mouse model – in which CRC is initiated with azoxymethane and promoted by repeated cycles of DSS administration (210) – with CSC exposure, revealed that CSC mice show accelerated development of macroscopic suspect lesions, as well as a trend toward an increased incidence of low- and/or high-grade colonic dysplasia (176). Although only a small fraction of these polyps may finally become malignant, there is evidence indicating that a large majority of colorectal carcinomas develop from these adenomatous polyps (211). Similarly, humans who develop severe dysplasia in adenomas are considered to be at increased risk of developing cancer (211). CSC mice further showed an increased number of Ki-67^+^ and a decreased number of TUNEL^+^ colonic epithelial cells, indicating abnormal patterns of cell replication, as detected in several clinical conditions associated with an increased risk for colorectal malignancies [for review see (212)]. A reduction in epithelial cell apoptosis already following 10 h of CSC (158), thereby, indicates that this effect is fast in onset and, hence, likely to be causally involved in CSC-induced promotion of azoxymethane/DSS-induced CRC. The latter was further indicated by increased colonic mRNA and/or protein expression of liver receptor homolog-1, β-catenin, cyclooxygenase II, and tumor necrosis factor alpha in CSC compared with SHC mice. Both liver receptor homolog-1 (213) and β-catenin (214) are involved in the control of intestinal cell renewal, and known to be involved in gastrointestinal tumor development (215–217). The same is true for cyclooxygenase II, which modulates apoptosis, angiogenesis, and tumor invasiveness [for review see (218)] and is over-expressed in approximately 80% of CRC and 40% of colorectal adenomas relative to normal mucosa (219). Tumor necrosis factor has been shown to promote signaling via the β-catenin pathway, thereby contributing to tumor development in the gastric mucosa (220).

Interestingly, a shift from protective Th cells to regulatory T cells was recently hypothesized to mediate the increased susceptibility of mice to UV-induced skin cancer following repeated immobilization (6 h/day over 3 weeks) (12). Similarly, increased regulatory T cell infiltration into the tumor bed, predicted reduced survival in cancer-bearing patients [for review see (221)]. Therefore, development of GC resistance in Th2-, but not Th1-, cell subpopulations during 19 days of CSC (169), causing a potential down-regulation of tumor protective Th1 immunity during repeated post-CSC DSS cycles (heterotypic immune stressors; for details see before), might be involved in CSC-induced CRC progression. An increased number of colonic CD4^+^ Th cells and percentage of CD3^+^ mesenteric lymph node cells in CSC versus SHC mice, but a decreased colonic interferon-γ mRNA expression coupled with an unaltered interferon-γ secretion from stimulated mesenteric lymph node cells support this hypothesis. An increased colonic FoxP3 mRNA expression, as well as number of CD3^+^/Foxp3^+^ double-positive mesenteric lymph node cells, following CSC further suggests enhanced immune regulation. Together with the above described reduction in regulatory T cell counts in peripheral lymph node tissue immediately following termination of CSC (169), these data either suggest tissue specificity of CSC effects or that regulatory T cell numbers normalize and even increase gradually following CSC, ameliorating CSC effects on subsequent inflammatory episodes (repeated DSS treatment) but promoting those on CRC development. Notably, body weight development of CSC and SHC mice during second and third DSS cycles is comparable, indicative of an equally severe colitis, whereas three CSC but no SHC mice were dying off too severe colitis during the first DSS cycle.

#### Affective disorders

##### Hyperactivity

Humans exposed to severe stressors are at increased risk for developing affective disorders, including post-traumatic stress disorder, which is characterized by pronounced and long-lasting hyperarousal, among other symptoms [for review see (222)]. A link between stress and hyperactivity is also suggested by studies revealing that prenatal stress in humans is associated with attention deficits, hyperarousal, and hyperactivity during childhood [(223); for review see (224)]. Poor school and social functioning, behavioral problems, and parental conflicts, all representing chronic psychosocial stressors, are further well-known factors predicting persistence of childhood attention deficit hyperactivity disorder into adolescence and adulthood (for review see 224). Moreover, bipolar disorder, which affects between 1–3% of the population, is characterized by a cycling between depressive episodes and periods of overactivity, termed mania. Given this strong overactivity component, it is not surprising that the majority of animal models used to study mania have focused on manipulations leading to hyperactivity, e.g., psychostimulant-induced hyperlocomotion [for review see (60, 225, 226)]. In contrast, studies employing animal models of repeated/chronic stress more or less consistently report a stress-induced reduction in locomotor activity, both in the homecage (33, 227) and in a novel environment during behavioral testing (67, 188, 200, 228, 229). Notably, while locomotor activity during behavioral testing (e.g., EPM) should ideally be dissociated from the anxiety state of the animal, altered locomotion is a general confound in such tests. For instance, reduced locomotion during EPM testing of rats following a single cat exposure might also be interpreted as reduced exploration due to increased levels of predator-induced anxiety-related behavior (230). Moreover, highly-anxious rodents (rats bred for high-anxiety-related behavior, HAB rats) show a gender-independent decrease in the number of line crossings in the dark compartment during LDB testing compared with their respective low anxious counterparts (rats bred for low-anxiety-related behavior, LAB rats) (231). Given that genotype specific differences in anxiety-related behavior between these breeding lines have been convincingly shown in locomotion-independent (i.e., ultrasound vocalization) anxiety tests, this indicates that reduced locomotion may be one characteristic of anxious animals [for review see (232)]. This is further indicated by the fact that locomotion in the open field has been used not only as an index of general locomotor activity or exploratory behavior but also as index of anxiety [as referenced in (233)].

Assessment of homecage locomotion before, immediately after, and 1 week after CSC stressor exposure (70) confirmed the expected increase in locomotor activity at the beginning of the dark phase in both SHC and CSC mice prior to stress. While this increase is not seen immediately following CSC exposure, it is even more pronounced in CSC versus SHC mice 1 week later, indicating a long-lasting induction of dark phase hyperlocomotion/hyperactivity. Thus, the CSC paradigm poses one of the few animal models, which might help unraveling the mechanisms underlying stress-promoted hyperactivity.

##### State anxiety

Chronic psychosocial stressors have also been shown to reliably increase state anxiety in rodents (66, 67, 188, 200, 228, 229, 234) and to be a risk factor for anxiety disorders in humans [for review see (58)]. In keeping, CSC results in a profound and robust increase in state anxiety, which has been confirmed in at least five independent behavioral tests. In detail, exposure to 19 days of CSC reduces the time spent on the open arms of an EPM (130, 131, 173), specifically their distal parts during open arm exposure (135), as well as the time spent in the lit compartment of a LDB (131, 175). Moreover, CSC mice enter the central zone of an OF arena less often, explore novel objects less intensely during a novel object test (46, 70), and spend less time in the outer zone of a platform during EPF exposure (142). Importantly, in a recent study, we further described that CSC mice spent less time on the open arms of an EPM 4 and 8 days after stressor termination, indicating that the stressor-induced change in emotionality is a long-lasting phenomenon (70). With regard to the potential influence of the CSC-induced hyperlocomotion on the interpretation of these tests, they were performed in the early light phase, when home-cage locomotion is not affected. Moreover, no difference in locomotion parameters, such as closed arm entries or distance traveled, were observed between SHC and CSC mice. Therefore, the anxiogenic effect of CSC is robust and long lasting.

Given the anxiogenic effect of CSC exposure and that individuals vary in their response to chronic stressor exposure (234–237), in a recent study, we tested whether the genetic predisposition for high versus low anxiety-related behavior determines the vulnerability to CSC. Interestingly, and in line with our hypothesis, HAB CD1 mice and CD1 mice not selected for anxiety-related behavior (NAB) are equally vulnerable to the CSC-induced behavioral, physiological, neuroendocrine, and immunological effects, whereas CD1 mice bred for low-anxiety-related behavior (LAB) are found to be stress resilient (41). The latter is indicated by the fact that all stress-related parameters, including anxiety-related behavior, are comparable between CSC and SHC mice in the LAB group. In contrast, in both HAB and NAB genotypes, CSC results in an increased adrenal weight, a reduced adrenal *in vitro* ACTH responsiveness substantiated by a lower plasma CORT:ACTH ratio, and an enhanced pro-inflammatory cytokine secretion from isolated and stimulated mesenteric lymph node cells compared with respective SHC mice. Notably, the CSC-induced increase in anxiety described before in C57BL/6 mice (as referenced above) was only detectable in the NAB group, probably due to a ceiling effect in the anxious HAB line.

##### Social anxiety

Social anxiety disorder with a lifetime prevalence of 12.1% (238), is the “persistent fear of one or more situations in which an individual is exposed to unfamiliar people or possible scrutiny by others.” People suffering from social anxiety disorder attempt to avoid social situations that they fear, which only lead to a persistence of the disorder (239). CSC does not appear to result in social anxiety, despite the profound increase in state anxiety. In more detail, CSC mice spend a similar time investigating a novel object (empty cage) and a social contact (cage with an unknown conspecific) during the SPAT on day 20 of CSC, indicating if anything a lack of social preference (70). Interestingly, they show less investigation in both contexts, suggesting that anxiety of the novel environment may be, at least in part, involved. However, unlike following chronic social defeat, CSC mice do not show active social avoidance, i.e., less time investigating the social context than the non-social context (234, 240–242). Furthermore, when assessed 1 week after stressor termination, despite their still anxious-like phenotype (less time investigating the empty cage), CSC mice prefer to explore the novel conspecific (70). It is important to note that these findings were obtained with non-familiar conspecifics, and it is possible, if indeed not likely, that CSC mice exposed to one of the residents that they faced during the CSC paradigm, would show active social avoidance. For example, acute social defeat has repeatedly been shown to lead to social avoidance, but only to the aggressor [(243, 244); for review see (245)]. In agreement with Kalueff and coworkers (246), the initial lack of social preference following CSC is likely to reflect a temporary social deficit rather than depressive-like behavior (234, 240–242), particularly as CSC mice do not display depression-related behavior in the other tests (70).

##### Depressive-like behavior

In the literature, the majority of social stress paradigms lead to both increased depression- and anxiety-related behavior (67, 200, 228, 234, 240). This is perhaps not surprising, as there is high co-morbidity between the two disorders (247–250). However, in order to really dissect the mechanisms underlying anxiety or depression, animal models are sorely warranted, which specifically induce one phenotype. Importantly, the CSC paradigm seems to represent such a model, given that it does not lead to deficits in anhedonia (saccharine-preference) or depressive-like behaviour in the FST or TST for at least 1 week following stressor-termination. The use of more than one behavioral test is important, as, for example, GABA_B_ receptor knockout mice were shown to display depression-related behavior in the FST, but not TST (251). Similarly, exposure to 10 days of social defeat did not alter FST or TST behavior, but lead to an anhedonic phenotype as assessed using the SPAT (234). Therefore, together with the SDR stress paradigm (252) and the social defeat/overcrowding stress paradigm (70, 199), CSC represents one of the few animal models that increase levels of anxiety without simultaneously increasing depression-related behavior. Of note, other depressive-like symptoms, such as cognitive dysfunction (63), have not yet been assessed following CSC.

##### Substance abuse disorders

Chronic psychosocial stress also represents a strong risk factor for the development of substance abuse disorders, such as alcoholism. Moreover, since CSC exposure reliably increases anxiety-related behavior (as referenced above), a known risk factor for developing ethanol- (EtOH) dependence in humans (253), we assessed whether CSC mice voluntarily consume more EtOH. Here, we could show that 14-day CSC exposure increases EtOH intake, as well as preference, without affecting taste preference or total fluid intake (175). This increased consumption is shown at all EtOH concentrations tested (2–8%), underlining the potency of CSC as a chronic stressor. This is in line with human studies, demonstrating a strong correlation between stressor exposure and the amount of EtOH consumed. It was, for instance, shown that individuals with increased numbers of stressful life events consume more EtOH and exhibit more indicators of EtOH dependence (254). In contrast, data gathered from rodent studies are less consistent. While, for instance, 5 min of daily social defeat over five consecutive days has the potential to increase EtOH consumption in male Long–Evans rats (255) and male C57BL/6 mice (256), there are also studies failing to detect a link between social stressor exposure and increased EtOH consumption [(257); for review see (258)]. These inconsistencies led us to consider our CSC model to be more relevant for the human situation, as it reliably induces an increase in EtOH consumption for a wide range of EtOH concentrations. At present, it is unclear whether CSC leads to abuse of other substances, such as cocaine or nicotine, remains to be determined in future studies.

##### Central mechanisms underlying the behavioral consequences of CSC

With respect to the central mechanisms underlying these CSC-induced behavioral consequences, we tested a possible involvement of the brain AVP, CRH, and OXT systems (130, 131, 155). These neuropeptides have all been linked with anxiety and substance abuse [(259–261); for review see (262)] and, thus, represent potential mediators of the CSC phenotype. While the expression patterns of hypothalamic OXT mRNA, generally known as an anxiolytic neuropeptide (38, 263–268), as well as the anxiogenic neuropeptide CRH (100) are not altered during CSC, the mRNA expression of the anxiogenic neuropeptide AVP (71, 269) is even reduced in the PVN following 20 days of CSC (131). Similarly, OXT mRNA expression in the PVN and supra optic nucleus is not affected on day 15 of CSC exposure (270). Immunohistochemistry further revealed unaffected numbers of AVP expressing parvo- and magnocellular PVN neurons in SHC and CSC mice (155), altogether making a substantial contribution of CRH, AVP, and OXT in CSC-induced anxiogenesis and EtOH preference, at least at the first glance, rather unlikely.

Recent findings strongly argue for a role of at least the oxytocinergic system in CSC-induced anxiety. Chronic central infusion of OXT (1 ng/h) via an osmotic minipump during 19-day CSC exposure – besides thymus atrophy, adrenal hypertrophy, and decreased adrenal *in vitro* ACTH sensitivity – further prevents CSC-induced anxiogenesis (270). The fact that chronic central OXT infusion additionally prevents the CSC-induced reduction in OXT receptor binding in the median raphe nucleus (270), a region in which OXT signaling has been recently implicated in serotonin release and subsequent anxiolytic effects (271), suggests a main role of the OXT system in this midbrain region in CSC-induced anxiety. Moreover, we have recent data showing that the same central OXT infusion procedure attenuates CSC-induced EtOH preference (Peters et al., unpublished observations). Since the raphe is hypothesized to be an important component of the circuitry involved in the reinforcing properties of drugs of abuse, including EtOH (272), and OXT can reduce drug intake and withdrawal symptoms (273), this region may be, at least in part, involved in CSC-induced heightened EtOH preference.

Besides these local changes in OXT-R binding, CSC and SHC mice show a different neuronal activity within various brain regions implicated in anxiety – under both basal and acute novel environment exposure conditions (135). For example, increased basal neuronal activation in the nucleus accumbens, as seen in CSC versus SHC, was shown in mice exposed to predator odor, which displayed increased anxiety-related behavior in the LDB (274). Furthermore, a decreased activation of the ventral and intermediate parts of the lateral septum, as seen in CSC mice following open arm exposure (135), has been described after acute stress in rats exposed to a learned-helplessness paradigm (275). Similarly, increasing the activity of lateral septum neurons was found to reduce feelings of fear and anxiety (276). Moreover, an increased activation of the dorsomedial part of the periaqueductal grey, as seen in open arm-exposed CSC versus SHC mice (135), has been reported in HAB rats after acute air jet exposure (277). Although acute stress-induced neuronal activation of the ventral hippocampus, a region well known to promote certain aspects of anxiety [for review see (278)], is not affected by CSC, given that cFOS activation in the hippocampal CA3 region is reduced in CSC versus SHC mice following open arm exposure (135). The latter may be explained by the well characterized effects of stress on retraction of dendritic spines (279), which is mainly restricted to this subfield (280). Of note, many of these changes occur in regions that form part of the reward circuitry, but whether acute administration of drugs of abuse (i.e., EtOH) would lead to differential activation between SHC versus CSC mice remains to be determined.

Together, these findings indicate that although the CSC-induced anxiety and substance abuse phenotype is pronounced, reliable, and long lasting, the detailed mechanisms behind are still poorly understood and await further investigation. Moreover, it remains to be seen whether traditional antidepressants or anxiolytics can reverse the CSC-induced behavioral and/or physiological phenotype. Furthermore, and more akin to the clinical situation, it will be interesting to assess whether post-CSC treatment of the mice can reverse the long-lasting behavioral and physiological consequences of stressor exposure.

## Conclusion

**Figure 3:**
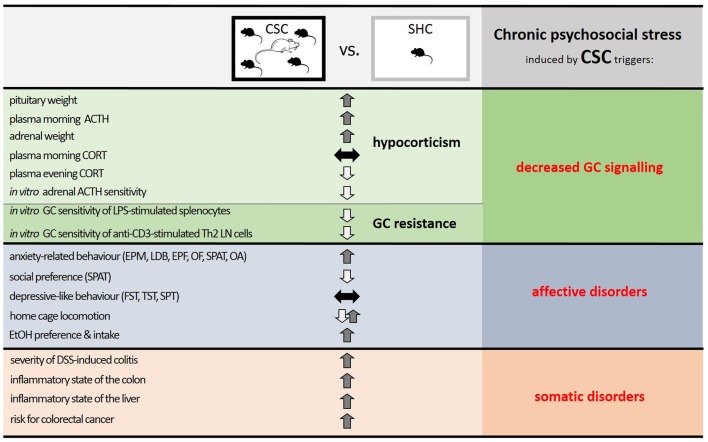
**Summary of the effects of chronic psychosocial stress in male mice induced by 19 days of chronic subordinate colony housing (CSC) on physiological, immunological, and behavioral parameters**. Compared with single-housed controls (SHC), CSC mice develop a decreased glucocorticoid (GC) signaling, induced by a combination of hypocorticism and GC resistance, and phenotypes characteristic of affective and somatic disorders. Given that stressors that can lead to somatic and affective disorders in humans are mainly chronic and psychosocial in nature, and result in a decreased GC signaling, the CSC paradigm represents a promising animal model to mimic stress-related pathologies in humans and to unravel the underlying mechanisms. Abbreviations: ACTH, adrenocorticotropic hormone; CORT, corticosterone; LPS, lipopolysaccharide; Th2, T helper 2; LN, lymph node; EPM, elevated plus-maze; LDB, light–dark box; EPF, elevated platform; OF, open field; SPAT, social preference/avoidance test; OA, open arm exposure; FST, forced swim test; TST, tail suspension test; SPT, saccharine preference test; EtOH, ethanol; DSS, dextran-sulfate sodium.

In this review, we have highlighted the fact that numerous somatic and affective disorders, for which chronic psychosocial stress is an accepted risk factor, are characterized by insufficient GC signaling. Thus, hypocorticism and/or GC resistance are observed in many disorders and following chronic psychosocial stress. Consequently, animal stress models, utilizing a chronic psychosocial component, which result in a decreased GC signaling and concomitant somatic and affective pathologies are likely to hold more translational relevance than other stress models. Indeed, chronic psychosocial stress in mice induced by the CSC paradigm results in both an anxiogenic and substance abuse phenotype, resembling affective disorders, and an overall pro-inflammatory- and cancer-prone phenotype, akin to somatic disorders. CSC further causes basal evening hypocorticism and GC resistance, resembling decreased GC signaling (see Figure 3).

Therefore, we are convinced that the CSC paradigm represents an appropriate animal model for studying stress-related disorders in which altered GC signaling is a core feature. Such detailed knowledge will provide further insight into how such stress-related HPA axis changes ultimately lead to somatic and affective disorders, at both behavioral and mechanistic level. Such detailed knowledge, in turn, will allow us to identify novel targets for the treatment of a wide variety of somatic and affective disorders.

## Conflict of Interest Statement

The Review Editor Karl Bechter declares that, despite being affiliated to the same institution as authors Dominik Langgartner, Andrea M. Füchsl and Stefan O. Reber, the review process was handled objectively and no conflict of interest exists. The authors declare that the research was conducted in the absence of any commercial or financial relationships that could be construed as a potential conflict of interest.
